# Supplementary material for: Accuracy of age estimation and assessment of the 18-year threshold based on second and third molar maturity in Koreans and Japanese
Source: PLoS One. 2022 Jul 8;17(7):e0271247. doi: 10.1371/journal.pone.0271247 (PMC9269881; doi:10.1371/journal.pone.0271247)
Supplement: S1 Table — (PDF) [file pone.0271247.s001.pdf]

S1 Table. Median and interquartile range of estimated ages according to chronologic age group.

Korean (DV)

	Male						Female					
Age group (years)	Min	Q1	Med	Q3	Max	IQR	Min	Q1	Med	Q3	Max	IQR
15	13.90	15.08	15.89	16.07	17.02	0.99	12.82	15.42	15.62	15.93	16.48	0.51
16	13.90	16.07	16.27	16.64	17.57	0.57	14.55	15.73	15.93	16.21	18.65	0.48
17	15.26	16.64	16.94	17.37	19.62	0.73	15.42	16.21	16.56	17.18	19.51	0.97
18	15.26	18.44	18.89	19.27	21.02	0.83	16.20	18.38	19.23	19.51	20.18	1.13
19	16.07	19.09	19.62	19.62	21.02	0.53	16.48	19.24	19.51	19.65	21.82	0.41
20	18.02	19.62	20.04	20.71	21.02	1.09	18.16	19.51	20.10	20.18	21.82	0.67
21	18.32	19.94	21.02	21.02	21.02	1.09	18.96	19.51	20.68	20.83	21.82	1.32
22	19.24	21.02	21.02	21.02	21.02	0.00	18.96	20.57	20.70	21.82	21.82	1.25
23	18.89	21.02	21.02	21.02	21.02	0.00	20.10	21.09	21.82	21.82	21.82	0.73

Japanese (DV)

	Male						Female					
Age group (years)	Min	Q1	Med	Q3	Max	IQR	Min	Q1	Med	Q3	Max	IQR
15	13.53	15.08	15.89	16.07	19.27	0.99	11.82	15.42	15.93	17.18	19.51	1.76
16	13.34	16.51	17.18	18.29	19.73	1.79	13.36	15.73	17.74	18.76	21.82	3.03
17	12.94	17.51	18.61	19.27	21.02	1.76	14.55	16.48	17.98	19.51	20.70	3.03
18	15.89	18.24	19.15	19.62	21.02	1.38	14.74	16.98	18.96	20.18	21.82	3.20
19	16.64	18.75	19.62	19.89	21.02	1.14	15.62	18.81	20.10	20.18	21.82	1.38
20	16.64	19.62	20.60	21.02	21.02	1.40	16.20	19.17	20.18	20.70	21.82	1.53
21	16.19	19.62	21.02	21.02	21.02	1.40	16.87	19.83	20.18	21.82	21.82	1.99
22	18.75	20.60	21.02	21.02	21.02	0.42	17.15	20.18	21.82	21.82	21.82	1.64
23	19.62	21.02	21.02	21.02	21.02	0.00	18.32	20.70	21.82	21.82	21.82	1.12

Korean (CV)

	Male						Female					
Age group (years)	Min	Q1	Med	Q3	Max	IQR	Min	Q1	Med	Q3	Max	IQR
15	14.22	15.36	15.86	16.24	17.62	0.88	13.12	15.62	15.93	16.36	17.10	0.74
16	14.36	16.24	16.62	17.12	18.00	0.88	15.19	15.93	16.36	16.79	18.55	0.86
17	15.48	17.09	17.12	17.62	19.80	0.53	15.62	16.79	17.19	17.62	19.60	0.83
18	15.74	18.42	18.92	19.42	20.68	1.00	16.67	18.72	19.17	19.60	20.34	0.88
19	16.24	19.04	19.80	19.80	20.68	0.76	17.10	19.29	19.60	19.91	21.08	0.62
20	18.16	19.80	20.18	20.40	20.68	0.59	18.43	19.60	20.03	20.34	21.08	0.74
21	18.04	20.09	20.68	20.68	20.68	0.59	18.86	19.60	20.68	20.65	21.08	1.05
22	19.30	20.68	20.68	20.68	20.68	0.00	18.86	20.43	20.65	21.08	21.08	0.65
23	18.92	20.68	20.68	20.68	20.68	0.00	20.03	20.65	21.08	21.08	21.08	0.43

Japanese (CV)

	Male						Female					
Age group (years)	Min	Q1	Med	Q3	Max	IQR	Min	Q1	Med	Q3	Max	IQR
15	13.56	15.36	15.86	16.24	19.42	0.88	12.38	15.62	16.36	17.17	19.60	1.55
16	12.74	16.68	17.28	18.42	19.80	1.74	13.93	16.05	17.81	18.79	21.08	2.74
17	13.44	17.66	18.71	19.42	20.68	1.76	15.10	17.10	18.43	19.60	20.65	2.50
18	15.86	18.26	19.04	19.80	20.68	1.54	15.10	17.41	19.08	20.34	21.08	2.93
19	17.12	18.98	19.80	19.99	20.68	1.01	16.05	18.60	20.03	20.34	21.08	1.74
20	17.12	19.80	20.30	20.68	20.68	0.88	16.67	19.28	20.34	20.65	21.08	1.37
21	16.62	19.80	20.68	20.68	20.68	0.88	17.31	19.79	20.34	21.08	21.08	1.29
22	19.04	20.30	20.68	20.68	20.68	0.38	17.84	20.34	21.08	21.08	21.08	0.74
23	19.80	20.68	20.68	20.68	20.68	0.00	18.77	20.65	21.08	21.08	21.08	0.43

Interquartile range is calculated by substracting Q1 from Q3. DV, discrete variable; CV, continuous variable; Min, minimum; Q1, first quartile; Med, median; Q3, third quartile; Max, maximum; IQR, interquartile range.
