# Supplementary material for: Accuracy of age estimation and assessment of the 18-year threshold based on second and third molar maturity in Koreans and Japanese
Source: PLoS One. 2022 Jul 8;17(7):e0271247. doi: 10.1371/journal.pone.0271247 (PMC9269881; doi:10.1371/journal.pone.0271247)
Supplement: S2 Table — (PDF) [file pone.0271247.s002.pdf]

S2 Table. Correlation coefficient between estimated and chronological age.

			Korean			Japanese		
			Male	Female	Total	Male	Female	Total
DV	UM2	PCC	0.79	0.77	0.77	0.61	0.48	0.54
		ICC	0.78	0.79	0.79	0.69	0.61	0.65
	UM3	PCC	0.80	0.78	0.79	0.74	0.62	0.66
		ICC	0.86	0.85	0.85	0.82	0.76	0.79
	LM2	PCC	0.76	0.80	0.78	0.57	0.48	0.52
		ICC	0.76	0.81	0.79	0.64	0.61	0.62
	LM3	PCC	0.82	0.77	0.79	0.77	0.69	0.71
		ICC	0.86	0.85	0.86	0.84	0.80	0.82
	UM2+LM2	PCC	0.81	0.83	0.82	0.66	0.54	0.60
		ICC	0.81	0.82	0.82	0.72	0.65	0.68
	UM3+LM3	PCC	0.84	0.81	0.82	0.78	0.7	0.72
		ICC	0.88	0.88	0.88	0.86	0.81	0.83
	UM2+UM3	PCC	0.88	0.88	0.88	0.77	0.66	0.71
		ICC	0.90	0.90	0.90	0.84	0.78	0.81
	LM2+LM3	PCC	0.88	0.89	0.88	0.78	0.69	0.72
		ICC	0.89	0.92	0.91	0.84	0.81	0.82

(a) Correlation coefficients using discrete variables. DV, discrete variable; CV, continuous variable; U, maxilla; L, mandible; Pearson correlation coefficients (PCCs) and interclass correlation coefficients (ICCs) were calculated based on age estimation using single tooth or combinations of two teeth. All point estimates are $P < 0.001$.

			Korean			Japanese		
			Male	Female	Total	Male	Female	Total
CV	UM2	PCC	0.79	0.76	0.78	0.60	0.48	0.54
		ICC	0.69	0.69	0.69	0.61	0.53	0.57
	UM3	PCC	0.83	0.83	0.82	0.77	0.69	0.71
		ICC	0.88	0.90	0.89	0.86	0.81	0.83
	LM2	PCC	0.76	0.80	0.78	0.57	0.48	0.52
		ICC	0.67	0.70	0.69	0.55	0.53	0.54
	LM3	PCC	0.83	0.81	0.82	0.79	0.73	0.74
		ICC	0.89	0.89	0.89	0.87	0.84	0.85
	UM2+UM3	PCC	0.81	0.82	0.81	0.65	0.54	0.59
		ICC	0.70	0.71	0.71	0.62	0.56	0.59
	UM2+LM2	PCC	0.86	0.85	0.85	0.81	0.74	0.76
		ICC	0.91	0.91	0.91	0.88	0.85	0.86
	UM3+LM3	PCC	0.88	0.88	0.88	0.75	0.66	0.70
		ICC	0.88	0.88	0.88	0.82	0.77	0.79
	LM2+LM3	PCC	0.87	0.89	0.88	0.78	0.71	0.74
		ICC	0.89	0.90	0.90	0.84	0.81	0.83

(b) Correlation coefficients using continuous variables. DV, discrete variable; CV, continuous variable; U, maxilla; L, mandible; Pearson correlation coefficients (PCCs) and interclass correlation coefficients (ICCs) were calculated based on age estimation using single tooth or combinations of two teeth. All point estimates are $P < 0.001$.
