# Supplementary material for: Accuracy of age estimation and assessment of the 18-year threshold based on second and third molar maturity in Koreans and Japanese
Source: PLoS One. 2022 Jul 8;17(7):e0271247. doi: 10.1371/journal.pone.0271247 (PMC9269881; doi:10.1371/journal.pone.0271247)
Supplement: S3 Table — (PDF) [file pone.0271247.s003.pdf]

S3 Table. Intercepts and coefficients of regression regarding the maturity stages of M2s and M3s as discrete and continuous variables for Korean data.

DV	Stg	Single tooth (Korean Male)				Single tooth (Korean Female)			
		UM2	UM3	LM2	LM3	UM2	UM3	LM2	LM3
Intercept		20.88	22.04	20.86	22.19	20.81	22.55	20.99	22.72
UM2	F (6)					-5.37			
	G (7)	-4.45				-4.29			
	H (8)	0				0			
UM3	B (2)		-5.15						
	C (3)		-6.43				-7.02		
	D (4)		-6.35				-6.47		
	E (5)		-5.18				-5.33		
	F (6)		-3.76				-3.30		
	G (7)		-2.43				-1.88		
	H (8)		0				0		
LM2	F (6)			-5.45				-5.74	
	G (7)			-4.28				-4.38	
	H (8)			0				0	
LM3	B (2)								-7.36
	C (3)				-6.60				-7.07
	D (4)				-6.41				-6.32
	E (5)				-5.48				-5.13
	F (6)				-4.28				-3.83
	G (7)				-2.12				-1.76
	H (8)				0				0
r^2		0.618	0.702	0.582	0.726	0.587	0.694	0.647	0.667

(a) Simple linear regression in Korean using discrete variables. The estimated age is calculated by adding an intercept and the numerical values equivalent in the stage of each tooth in each column. DV, discrete variable; CV, continuous variable; U, maxilla; L, mandible; Stg, stage; Stages 1 to 8 indicate the scores of Demirjian developmental stages A to H. All *P*-values are less than 0.05 ($P < 0.05$).

DV	Stg	Two teeth (Korean Male)				Two teeth (Korean Female)			
		UM2+ LM2	UM3+ LM3	UM2+ UM3	LM2+ LM3	UM2+ LM2	UM3+ LM3	UM2+ UM3	LM2+ LM3
Intercept		21.00	22.33	22.05	22.19	21.07	22.89	22.55	22.72
UM2	F (6)					-1.52*		-3.07	
	G (7)	-2.78		-2.43		-1.91		-2.43	
	H (8)	0		0		0		0	
UM3	B (2)		-1.57*	-2.73					
	C (3)		-3.07	-4.01			-4.16	-4.59	
	D (4)		-2.96	-4.01			-3.91	-4.34	
	E (5)		-2.37	-3.41			-3.24	-3.74	
	F (6)		-1.81	-2.63			-1.83	-2.74	
	G (7)		-1.33	-2.11			-1.13	-1.71	
	H (8)		0	0			0	0	
LM2	F (6)	-2.81			-2.11	-4.11			-3.47
	G (7)	-1.97			-2.02	-2.90			-2.75
	H (8)	0			0	0			0
LM3	B (2)						-3.83		-4.26
	C (3)		-3.80		-4.70		-3.43		-4.32
	D (4)		-3.87		-4.64		-2.97		-3.91
	E (5)		-3.28		-3.99		-2.39		-3.35
	F (6)		-2.60		-3.10		-2.00		-2.88
	G (7)		-1.21		-1.91		-0.91		-1.59
	H (8)		0		0		0		0
adj. r^2		0.655	0.762	0.796	0.792	0.688	0.733	0.803	0.812

(b) Multiple linear regression in Korean using discrete variables. The estimated age is calculated by adding an intercept and the numerical values equivalent in the stage of each tooth in each column. DV, discrete variable; CV, continuous variable; U, maxilla; L, mandible; Stg, stage; Stages 1 to 8 indicate the scores of Demirjian developmental stages A to H. $^{*}P > 0.05$, not statistically significant.

	Single tooth (Korean Male)				Single tooth (Korean Female)			
CV	UM2	UM3	LM2	LM3	UM2	UM3	LM2	LM3
Intercept	-14.69	8.77	-12.60	8.38	-13.11	9.23	-13.30	9.80
UM2	4.45				4.24			
UM3		1.62				1.66		
LM2			4.18				4.28	
LM3				1.69				1.58
r^2	0.618	0.681	0.577	0.695	0.584	0.688	0.642	0.654

	Two teeth (Korean Male)				Two teeth (Korean Female)			
CV	UM2+ LM2	UM3+ LM3	UM2+ UM3	LM2+ LM3	UM2+ LM2	UM3+ LM3	UM2+ UM3	LM2+ LM3
Intercept	-16.81	7.85	-5.43	-3.53	-16.64	8.70	-5.05	-6.05
UM2	2.83		2.33		1.89		2.26	
UM3		0.80	1.06			1.02	1.15	
LM2	1.89			1.98	2.82			2.56
LM3		0.96		1.19		0.72		0.98
adj. r^2	0.655	0.734	0.769	0.765	0.683	0.725	0.791	0.787

(c) Simple and multiple linear regression in Korean using continuous variables. The estimated age is calculated by adding an intercept to the multiply of the stage of each tooth (1-8) and the numerical value on each column. DV, discrete variable; CV, continuous variable; U, maxilla; L, mandible; All *P*-values are less than 0.05 ($P < 0.05$).
