# Supplementary material for: Accuracy of age estimation and assessment of the 18-year threshold based on second and third molar maturity in Koreans and Japanese
Source: PLoS One. 2022 Jul 8;17(7):e0271247. doi: 10.1371/journal.pone.0271247 (PMC9269881; doi:10.1371/journal.pone.0271247)
Supplement: S1 Fig — (PDF) [file pone.0271247.s005.pdf]

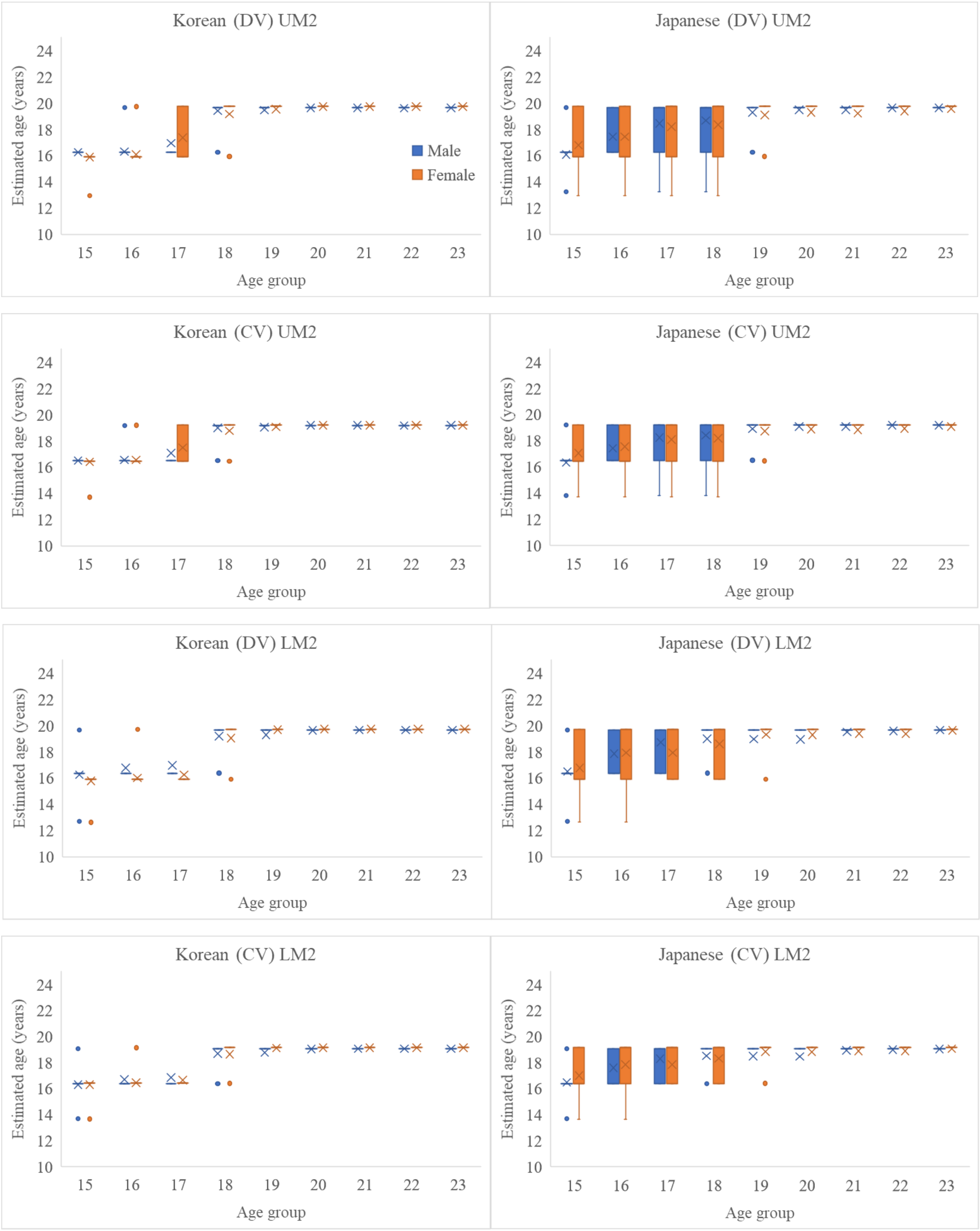

S1 Fig. Box plots for comparison between chronological and estimated ages. (a) The age was estimated based on the maturity of single tooth (UM2 or LM2), and analyses were performed based on the assumption that the variables were discrete and continuous. DV, discrete variable; CV, continuous variable; U, maxilla; L, mandible; Blue represents male and orange represents female.

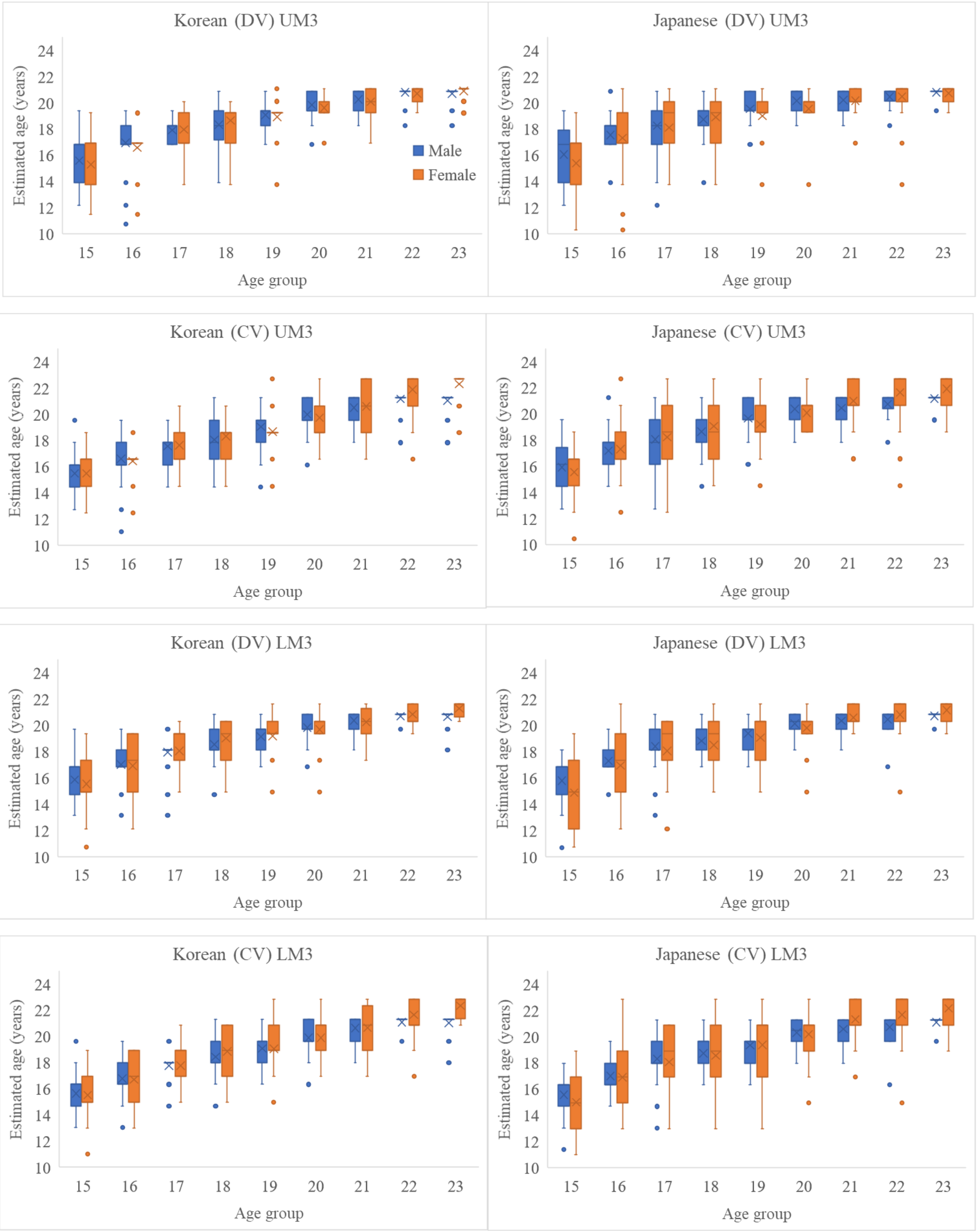

(b) The age was estimated based on the maturity of single tooth (UM3 or LM3), and analyses were performed based on the assumption that the variables were discrete and continuous. DV, discrete variable; CV, continuous variable; U, maxilla; L, mandible; Blue represents male and orange represents female.

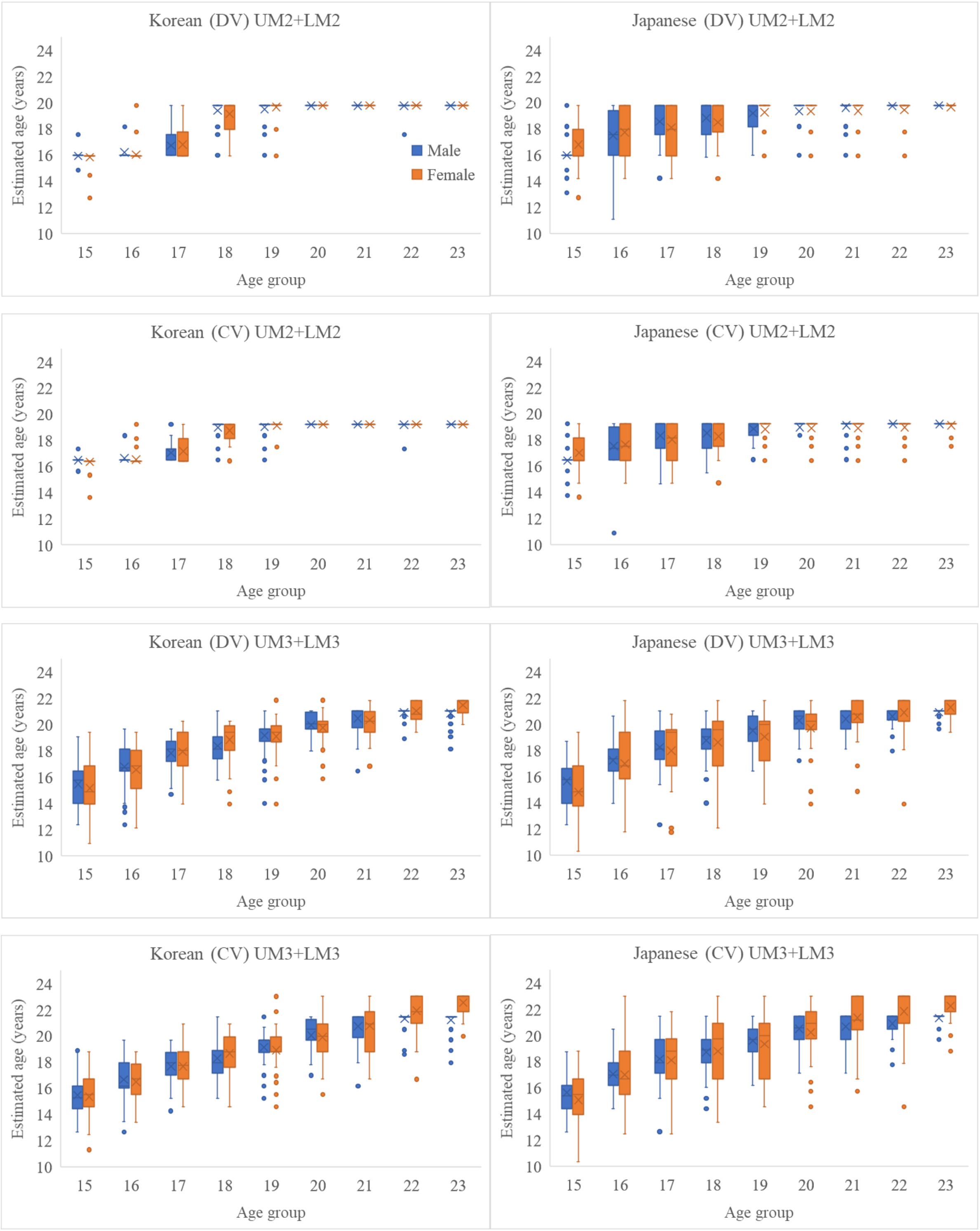

(c) The age was estimated based on the maturity of two teeth (UM2+LM2 or UM3+LM3), and analyses were performed based on the assumption that the variables were discrete and continuous. DV, discrete variable; CV, continuous variable; U, maxilla; L, mandible; Blue represents male and orange represents female.

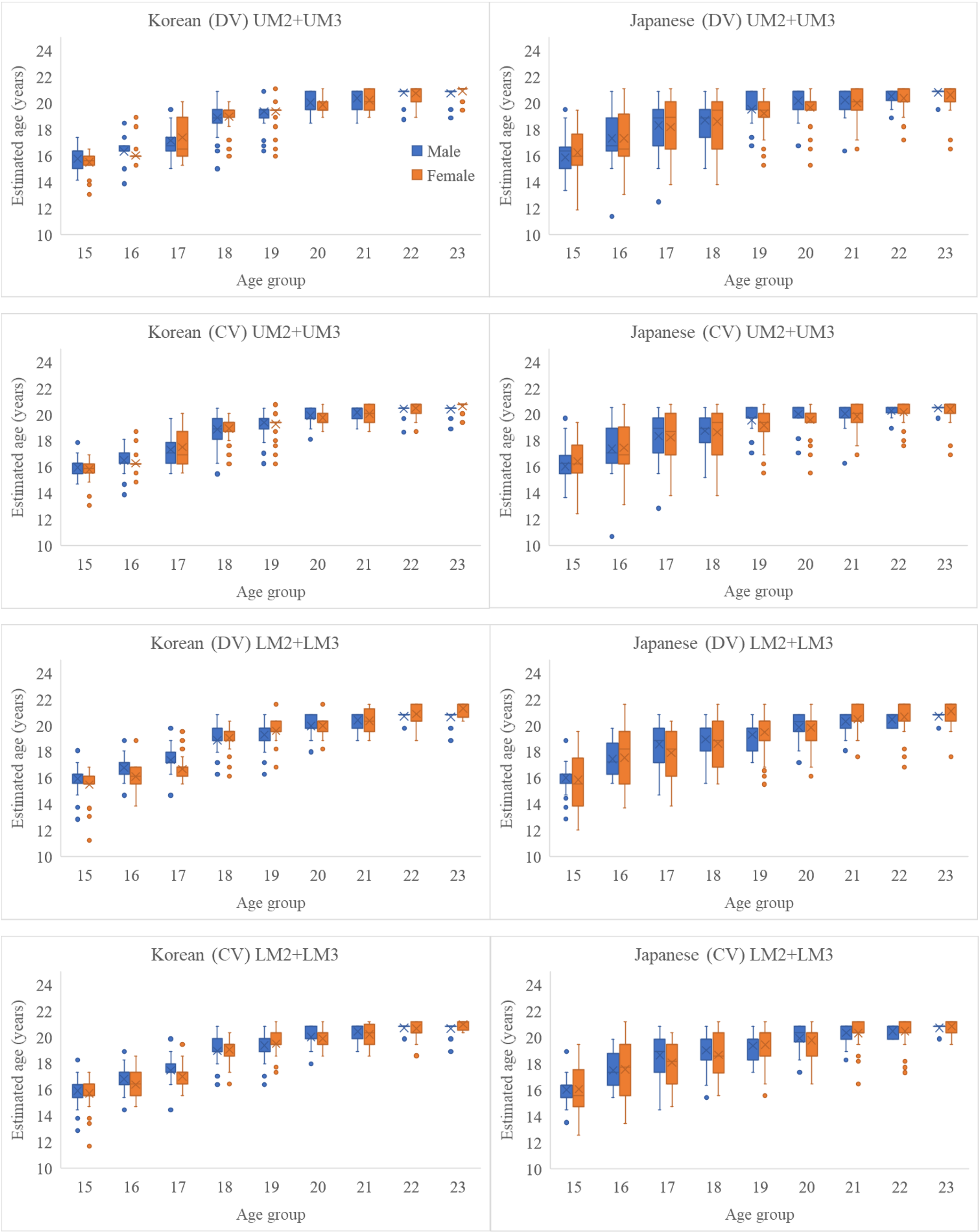

(d) The age was estimated based on the maturity of two teeth (UM2+UM3 or LM2+LM3), and analyses were performed based on the assumption that the variables were discrete and continuous. DV, discrete variable; CV, continuous variable; U, maxilla; L, mandible; Blue represents male and orange represents female.
