# Supplementary material for: Accuracy of age estimation and assessment of the 18-year threshold based on second and third molar maturity in Koreans and Japanese
Source: PLoS One. 2022 Jul 8;17(7):e0271247. doi: 10.1371/journal.pone.0271247 (PMC9269881; doi:10.1371/journal.pone.0271247)
Supplement: Attachment — Submitted filename: Response to reviewer.docx [file pone.0271247.s007.docx]

**Respond to editor**

**#1.** Please submit your revised manuscript by Jul 30 2022 11:59PM. If you will need more time than this to complete your revisions, please reply to this message or contact the journal office at plosone@plos.org. When you're ready to submit your revision, log on to https://www.editorialmanager.com/pone/ and select the 'Submissions Needing Revision' folder to locate your manuscript file.

**- Response:** It is already done. Thank you.

**#2.** Please include the following items when submitting your revised manuscript:

A rebuttal letter that responds to each point raised by the academic editor and reviewer(s). You should upload this letter as a separate file labeled 'Response to Reviewers'.

A marked-up copy of your manuscript that highlights changes made to the original version. You should upload this as a separate file labeled 'Revised Manuscript with Track Changes'.

An unmarked version of your revised paper without tracked changes. You should upload this as a separate file labeled 'Manuscript'.

**- Response:** All three files were checked and uploaded. Thank you.

**#3.** If you would like to make changes to your financial disclosure, please include your updated statement in your cover letter.

**- Response:** We also uploaded the updated “cover letter” which was indicating the changes in financial disclosure. Thank you so much. The underlined contents were added in financial disclosure.

**- Corrections**: This research was supported by Catholic Medical Center Research Foundation (SSL, 5-2021-B0001-00309) funded by The Catholic University of Korea and this work was also supported by National Forensic Service (SSL, NFS2022MED08), Ministry of the Interior and Safety, Republic of Korea. The funders had no role in study design, data collection and analysis, decision to publish, or preparation of the manuscript.

**#4.** Guidelines for resubmitting your figure files are available below the reviewer comments at the end of this letter.

**- Response:** There is no changes in figure files. Thank you.

**#5.** If applicable, we recommend that you deposit your laboratory protocols in protocols.io to enhance the reproducibility of your results. Protocols.io assigns your protocol its own identifier (DOI) so that it can be cited independently in the future. For instructions see: https://journals.plos.org/plosone/s/submission-guidelines#loc-laboratory-protocols. Additionally, PLOS ONE offers an option for publishing peer-reviewed Lab Protocol articles, which describe protocols hosted on protocols.io. Read more information on sharing protocols at <https://plos.org/protocols?utm_medium=editorial-email&utm_source=authorletters&utm_campaign=protocols>.

**- Response:** There is no lab protocols in this study. Thank you.

**#6.** Please review your reference list to ensure that it is complete and correct. If you have cited papers that have been retracted, please include the rationale for doing so in the manuscript text or remove these references and replace them with relevant current references. Any changes to the reference list should be mentioned in the rebuttal letter that accompanies your revised manuscript. If you need to cite a retracted article, indicate the article’s retracted status in the References list and also include a citation and full reference for the retraction notice.

**- Response:** We checked the reference list again, there was no retracted article, and we confirmed the reference list in this manuscript was complete and correct. Thank you so much.

**Respond to reviewer**

**Reviewer: 1**

**#1.** I kindly request the authors to look at their sample size calculation and sampling method, better if they consult statistician. I'm asking how do you reach into your final sample size? they said the minimum sample size was calculated as 403 but the final sample size was 2657, what is this miss much? How you withdraw your final samples from the source population?

**- Response:** With the opinion from reviewer #1, we checked our manuscript again, and found the expression might be rise the misunderstanding. The sample size was calculated as 403 for the single regression model. In this study, the regression model was performed on a population stratified by gender and nation. Therefore, the sample size which we presented was over 403. In our opinion, we used only the minimum sample size for each regression model and the total samples we analyzed was 2657. Nevertheless, we corrected sentences that may cause misunderstandings by the readers. Thank you for your suggestion. It can make this manuscript clearer for readers.

**- Corrections**: Data were randomly selected and stratified by gender and population from the radiographs which were enrolled from Seoul St. Mary’s Hospital, Catholic University of Korea (900 males and 900 females, 19.47±2.62 years), and Iwate Medical University of Japan (406 males and 451 females, 19.31±2.60 years). (Line 101~104)
